# Enteric viral infections promote systemic accelerated aging in *Drosophila*

**DOI:** 10.1126/sciadv.aec1735

**Published:** 2026-06-05

**Authors:** Rubén González, Mauro Castelló-Sanjuán, Maria-Carla Saleh

**Affiliations:** Institut Pasteur, Viruses and RNA Interference Unit, Université Paris Cité, Paris, France.

## Abstract

Do viral infections accelerate aging, and does this acceleration scale with pathogenicity? Using transcriptomic aging clocks, we measured biological age in *Drosophila melanogaster* infected with four enteric RNA viruses spanning a broad pathogenicity range (i.e., reduction of host lifespan). All pathogenic infections accelerated aging and the magnitude of acceleration tracked pathogenicity. This pattern held across oral and systemic infection routes and was conserved in *Caenorhabditis elegans* where the nonpathogenic Orsay virus produced negligible aging acceleration. Pathway analysis indicated a systemic impact across aging hallmarks with virus- and tissue-specific signatures. Acceleration was comparable in females and males, but host context modulated the acceleration: The bacterial symbiont *Wolbachia* mitigated the virus-induced aging. Notably, biological age remained elevated even after viral clearance. These results demonstrate that viruses act as age-distorters and link infection severity to lasting aging consequences, providing a quantitative framework for predicting long-term health effects of viral disease.

## Teaser

Enteric viruses act as age-distorters, with infection severity tied to aging hallmarks and consequences.

## INTRODUCTION

Aging is a complex biological process characterized by progressive functional decline and increased vulnerability to disease and death (*1*). While chronological age measures time since birth, biological age reflects the functional status of an organism and can deviate notably from chronological age depending on genetic, environmental, and pathological factors (*2*–*5*). Understanding the mechanisms that accelerate or decelerate aging has profound implications for health and longevity.

Among the various factors that may influence aging, viral infections have emerged as potential modulators of the aging process. Infections with human cytomegalovirus, SARS-CoV-2, HIV, and hepatitis viruses drive premature aging through sustained inflammation, immune system exhaustion, and persistent cellular stress responses (*6*–*9*). These observations raise fundamental questions about whether viral infections truly accelerate aging processes beyond their direct pathogenic effects.

Quantifying aging acceleration requires robust biological age estimation methods: Cellular senescence assays, metabolomic profiling, and DNA methylation patterns (*10*–*14*) allow for estimation of various hallmarks of aging including genomic instability, telomere attrition, cellular senescence, and metabolic dysfunction (*15*, *16*). Currently, state-of-the-art computational methods combined with omics data enable the construction of “aging clocks” that capture the molecular complexity of aging across multiple biological layers (*17*). These clocks leverage machine learning approaches to integrate thousands of molecular features into composite measures of biological age, with different omics modalities (methylation, transcriptomics, and proteomics) often capturing distinct and complementary aspects of aging biology. Transcriptional aging clocks, in particular, leverage genome-wide expression data to capture aging signatures and estimate biological age (*18*–*20*).

The fruit fly *Drosophila melanogaster* is an ideal model organism for studying aging. Flies have a short lifespan, minimal ethical research concerns, abundant research resources available, and share a large number of functional homologies with humans (*21*–*26*). Specifically, the evolutionary conservation of aging mechanisms between flies and humans extends to over 70% of genes associated with aging-related diseases (*27*, *28*). In addition, fly aging involves multiple hallmarks conserved with humans such as genomic instability, telomere dysfunction, epigenetic alterations, loss of proteostasis, dysbiosis, mitochondrial dysfunction, and stem cell exhaustion (*29*, *30*).

At the tissue level, fly organs also share similarities with mammals with the fly intestine emerging as a particularly critical determinant of fly lifespan. *Drosophila* intestinal infection and pathology serve as an established model for human intestinal diseases and inflammatory conditions (*31*). Aged intestines progressively lose barrier function, develop dysbiosis, and exhibit stem cell overproliferation. These changes precede death and correlate strongly with survival (*30*, *32*–*36*).

Viral infections in *Drosophila* can elicit phenotypes characteristic of uninfected aged flies, including intestinal stem cell (ISC) hyperproliferation and barrier dysfunction (*37*, *38*). Because wild flies naturally harbor diverse RNA viruses with varying virulence (*39*, *40*), we focused on mono-infections of four enteric positive-sense RNA viruses—Bloomfield virus, Drosophila A virus (DAV), Drosophila C virus (DCV), and Nora virus—to test whether infection accelerates aging and whether the magnitude of this effect scales with pathogenicity. These viruses establish persistent fecal-oral infections from larval stages into adulthood, initially targeting the intestine before spreading systemically. We used transcriptional aging clocks to quantify biological age and to identify mechanisms and host factors that drive infection-induced age acceleration.

## RESULTS

### Persistent viral infections reduce fly lifespan and induce distinct transcriptional responses

As a system for studying infection-aging relationships, we used persistently mono-infected populations of *D. melanogaster*. These infections were established through oral acquisition of the virus during the larval stage from virus-contaminated environments, simulating natural environmental exposure patterns. Infection occurs upon larval hatching, with virtually all individuals maintaining persistent infections throughout their development and adult lifespan (*41*). This approach captures the chronic, long-term effects of persistent infection rather than acute pathological responses.

**Fig. 1.**
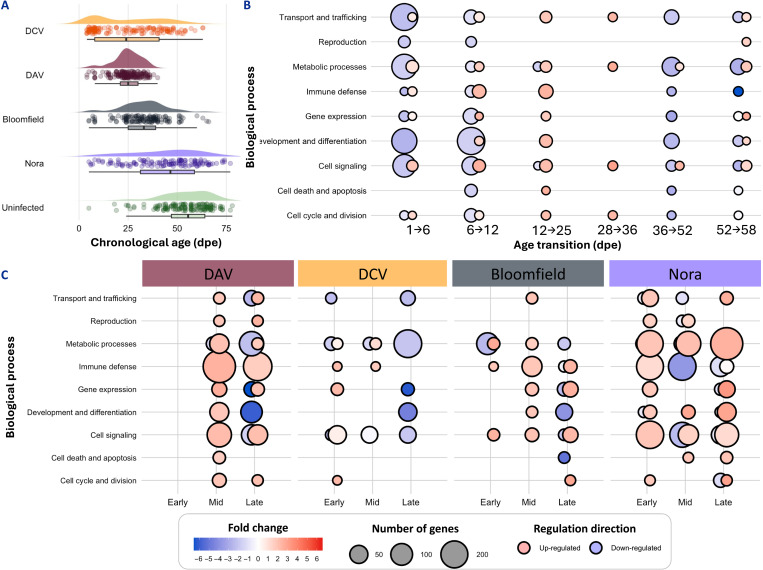
Characterization of viral pathogenicity and associated transcriptional responses. (**A**) Persistent viral infections reduce fly lifespan. Half-violin plot shows the distribution of flies lifespan, with each dot representing the day of death of individual flies and box plots summarizing the data per condition. (**B**) Aging in uninfected flies involves coordinated transcriptional changes across multiple biological processes. Circular symbols show the regulation of biological processes during consecutive age transitions in uninfected flies, comparing early to late-life time points (6 versus 1, 12 versus 6, 25 versus 12, and 52 versus 36 dpe). Red indicates up-regulation, and blue indicates down-regulation. Symbol size represents the number of genes affected, and color intensity indicates the magnitude of fold change. (**C**) Viral infections induce distinct transcriptional responses with virus-specific patterns. Circular symbols show transcriptional regulation in virus-infected flies compared to age-matched uninfected controls at three infection stages. Each virus shows unique temporal patterns of transcriptional regulation across infection stages. Early: 1 dpe; mid: 12 dpe; late corresponds to 50% mortality timing for each virus: 25 dpe for DAV, 28 dpe for DCV, 36 dpe for Bloomfield virus, and 52 dpe for Nora virus. Symbol conventions as in (B).

Lifespan analysis of infected *Drosophila* revealed virus-specific mortality patterns (Fig. 1A). Uninfected flies had a median lifespan of 55.5 ± 12.7 days post adult eclosion (dpe; median ± SD), while infected flies showed significantly reduced lifespans. The most severe effects occurred with DAV (25.0 ± 7.2 dpe) and DCV (24.0 ± 17.1 dpe), representing approximately 55% reduction compared to controls. Bloomfield virus caused intermediate mortality (33.0 ± 11.9 dpe, 40% reduction), while Nora virus showed the mildest impact (46.5 ± 17.9 dpe, 16% reduction).

To understand the molecular basis of these differences in lifespan, we characterized transcriptional changes across time in uninfected and infected flies. Analysis of uninfected flies across six age transitions revealed coordinated expression changes in multiple biological processes (Fig. 1B and file S1). Early-life time points (1 to 6 dpe) involved moderate transcriptional changes affecting metabolic processes and development. Mid-life time points (6 to 12 and 12 to 25 dpe) showed extensive transcriptional remodeling, with up-regulation of immune defense pathways and regulation of metabolic and signaling processes. Late-life time points (36 to 52 and 52 to 58 dpe) were characterized by declining expression of genes involved in metabolism, development, and cellular maintenance.

Viral infections induced distinct transcriptional responses that were virus-specific (Fig. 1C and file S1). DAV and Nora virus caused widespread transcriptional changes from early to late infection, while DCV and Bloomfield virus elicited more moderate responses. Both highly lethal DAV and mildly lethal Nora virus infections induced extensive transcriptional changes, but they affected different biological processes and followed different temporal patterns. This observation suggested that the relationship between transcriptional responses and aging acceleration might be more complex than simple magnitude of gene expression changes, motivating our subsequent quantitative analysis and the use of computational aging clocks to directly measure biological age acceleration rather than relying on transcriptional similarity as a proxy for aging.

### Transcriptional aging clocks reveal virus-induced aging acceleration

To investigate whether the reduced lifespan was accompanied by acceleration of aging, we employed RAPToR (*19*) to calculate the biological age from gene expression data (*41*).

The existing *Drosophila* aging reference accompanying RAPToR had notable limitations for our study. Most critically, the available reference relies on a two-decade-old microarray dataset (*42*) prepared from flies of unknown viral infection status, creating data compatibility issues with modern RNA sequencing (RNA-seq) outputs, and potentially introducing viral contamination artifacts. We therefore constructed a custom aging reference optimized for our experimental conditions. This approach provided critical advantages: confirmed viral infection–free reference samples, RNA-seq data compatibility (avoiding microarray–to–RNA-seq conversion artifacts), higher temporal resolution, and matched experimental conditions. These factors collectively optimized RAPToR performance. We used whole-genome transcriptional data from RNA-seq confirmed virus-free flies collected at eight time points (1, 6, 12, 25, 28, 36, 52, and 58 dpe) from our previous work (*41*). RAPToR’s interpolation capabilities allow the method to reliably estimate biological ages both within and beyond the original reference time points (*19*).

The resulting aging reference accurately captured uninfected fly aging dynamics. When applied to the same uninfected flies used for reference construction, biological age estimates showed strong correlation with chronological age (Pearson’s *r* = 0.997, *P* < 0.001) (fig. S1A). Leave-one-out cross-validation, when predicting the age of independent samples not used in reference construction, confirmed the accuracy and generalizability of our aging clock (Pearson’s *r* = 0.935, *P* < 0.001) (fig. S1B). To independently validate our aging acceleration metric, we leveraged a gene set from Scott *et al.* (*43*), who identified 328 genes that correlate with chronological age during the terminal decline phase in *Drosophila*. Principal components analysis (PCA) of these genes in our virus-infected samples revealed distinct transcriptional profiles across viruses and time points (fig. S2A). Notably, the first principal component (PC1), capturing the primary axis of variation in this dying-phase signature, strongly correlated with our RAPToR-based aging acceleration (Pearson’s *r* = 0.880; fig. S2B). This concordance confirms that our cumulative aging metric captures late-life transcriptional changes independently identified in naturally aging flies.

To further validate that our aging clock captures physiological frailty and mortality risk, we examined the “Smurf” phenotype using an independent dataset. The Smurf assay is a noninvasive method that detects intestinal barrier dysfunction by feeding flies a nontoxic blue dye; when barrier integrity is compromised, the dye spreads throughout the body, turning flies blue (*34*). The Smurf phenotype marks the terminal predeath phase, identifying frail individuals with high mortality risk: flies typically die within a few days of becoming Smurf regardless of their chronological age (*34*, *44*). Previous results revealed that persistent viral infections accelerate the appearance of this phenotype in *Drosophila*, with infected flies exhibiting reduced time to Smurf compared to uninfected controls (*37*). We analyzed transcriptomic data from uninfected flies stratified by Smurf status at matched chronological ages (*44*). At three chronological ages (20, 30, and 40 dpe), non-Smurf flies maintained biological ages close to their chronological ages. In contrast, chronological age-matched Smurf flies showed markedly accelerated biological aging at all time points (fig. S3). This demonstrates that our transcriptional aging clock captures physiological frailty states and validates that transcriptional aging estimates align with established aging phenotypes.

**Fig. 2.**
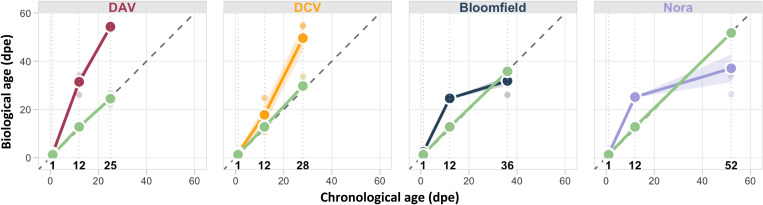
Viral infections disrupt fly aging. Panels show RAPToR-estimated biological age (*Y* axis) plotted against chronological age (*X* axis) for four different viral infections compared to uninfected controls. Time points include an early stage at 1 dpe, an intermediate stage at 12 dpe, and later stages corresponding to 50% mortality of the host population (25 dpe for DAV, 28 dpe for DCV, 36 dpe for Bloomfield virus, and 52 dpe for Nora virus). Colored lines represent virus-infected flies (DAV in red, DCV in orange, Bloomfield virus in blue, and Nora virus in purple), while green lines represent uninfected controls. Large points represent the mean biological age of four biological replicates per condition, and shaded areas represent SE. The dashed diagonal line indicates perfect correlation between chronological and biological age.

With this validated aging clock, we assessed the biological age of virus-infected flies at three time points: early stage at 1 dpe, intermediate stage at 12 dpe, and a later stage corresponding to 50% mortality of the host population (25 dpe for DAV, 28 dpe for DCV, 36 dpe for Bloomfield virus, and 52 dpe for Nora virus) (Fig. 2 and fig. S4). At 1 dpe, the biological age of uninfected animals was 1.2 ± 0.4 dpe (mean ± SD). Flies infected with Nora virus did not show a significant increase in aging (1.6 ± 0.2 days), but other viruses exhibited accelerated aging even at this early stage: 2.36 ± 0.2 dpe for Bloomfield virus, 1.8 ± 0.3 dpe for DAV, and 2.0 ± 0.2 dpe for DCV. At 12 dpe, the mean biological age of uninfected flies remained similar to their chronological age (12.8 ± 0.9 days), whereas all viral infections significantly increased the biological age: 31.5 ± 3.7 dpe for DAV, 17.8 ± 8.1 dpe for DCV, 24.6 ± 0.3 dpe for Bloomfield, and 25.2 ± 0.6 dpe for Nora. At later time points, DAV-infected flies had a biological age of 54.3 ± 0.1 dpe at 25 dpe and DCV-infected flies had a biological age of 49.6 ± 10.4 dpe at 28 dpe. For the other viruses, there was no accelerated aging at later time points; Bloomfield virus–infected flies showed a reduced but nonsignificant difference in biological age (31.9 ± 3.9 dpe at 36 dpe), and Nora virus–infected flies exhibited a significant slowing of aging (biological age of 37.1 ± 11.5 dpe at 53 dpe). These results demonstrate that enteric viral infections accelerate aging in a virus-specific manner. We previously showed that all four viruses induce ISC hyperproliferation at 20 days posteclosion, a canonical *Drosophila* aging phenotype [see figure S1 (F and G) in Nigg *et al.* (*37*)]. ISC hyperproliferation levels are proportional to both viral pathogenicity and RAPToR-detected aging acceleration, validating that our transcriptomic measurements capture physiological aging processes.

### Virus-induced accelerated aging is systemic, temporally dynamic, and targets aging pathways and tissues in a virus-specific manner

We investigated whether the observed aging acceleration reflects broad systemic effects (affecting multiple tissues and pathways) or arises from modulation of specific pathways. To do so, we created specialized aging references using genes associated with key aging processes (such as telomere shortening, stem cell dysfunction, and protein damage) and major tissues (gut, fat body, muscle, brain, and ovary). We then assessed acceleration of individual aging processes by estimating biological age to chronological age ratios for each process using our specialized references at the three critical time points described in the previous section: early, mid, and late life. Aging pathways were established from proved aging hallmarks (*15*) and curated from FlyBase pathways, and tissue-specific genes were obtained from FlyBase tissue enrichment data (*26*). Complete gene lists and identifiers can be found in file S2.

**Fig. 3.**
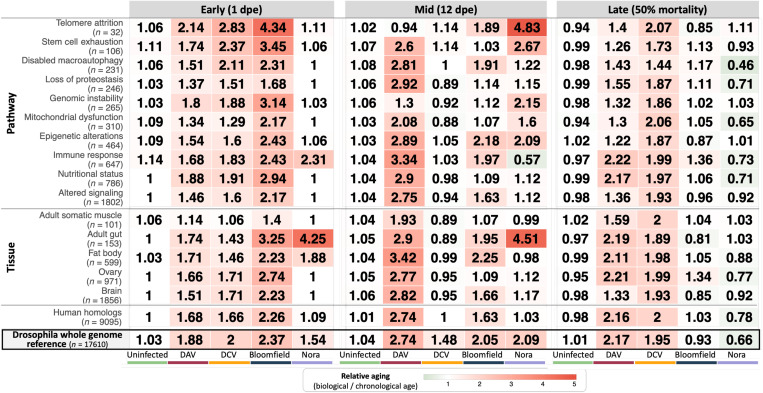
Temporal dynamics of virus-specific targeting of aging pathways and tissues. Heatmap shows median relative aging acceleration (biological age/chronological age) across infection progression for tissue-specific and pathway-specific aging references. Early life (1 dpe), mid-life (12 dpe), and late-life time points (DAV: 25 dpe, DCV: 28 dpe, Bloomfield: 36 dpe, and Nora: 52 dpe, representing ~50% population mortality). For uninfected flies, the late time point represents the median across samples from 25, 28, 36, and 52 dpe. Values >1.0 (red) indicate accelerated aging, values <1.0 (green) indicate slowed aging, and values 1.0 (white) indicate normal aging progression. Numbers in parentheses indicate the number of genes in each reference set. Median absolute deviation values provided in file S1.

Temporal analysis revealed distinct patterns of virus-induced aging acceleration that diverge from normal aging trajectories (Fig. 3). Uninfected flies maintained median biological to chronological age ratios close to 1 across all time points and tissue-specific references, demonstrating that our aging references accurately estimated aging in all organs and that hallmarks of aging pathways alone could reliably predict biological age. This established the baseline for normal aging progression. In contrast, viral infections accelerated aging from the very first day of adult life (early life time point), with most virus-pathway combinations showing elevated aging ratios immediately upon adult eclosion. This acceleration was maintained through the mid-life time point (12 dpe), indicating sustained aging pressure during active viral infection phases. Late-life time points revealed divergence between viruses. DAV and DCV maintained a high aging acceleration into late infection, with many pathways showing continued accelerated aging effects. Bloomfield virus showed broad pathway targeting that decreased over time, with aging pace returning to the levels seen in uninfected flies, indicating resolution of aging effects despite continued infection. However, Nora virus showed a pattern where aging effects were broad only at the mid–time point, with late-stage aging falling below the biological to chronological age ratio of uninfected controls, suggesting that infected flies that survived until this late stage had deployed tolerance mechanisms that slowed their aging below normal rates.

Analysis of tissue-specific aging acceleration revealed that all tissues tested showed evidence of accelerated aging at some time points, confirming the systemic nature of virus-induced aging effects. However, the adult gut and fat body emerged as the most critical targets of virus-induced aging across all viruses and time points, and in the case of the Nora virus infected individuals, were the only tissues with accelerated aging. Among aging pathways, immune response and telomere attrition showed elevated aging ratios in most conditions and time points. Beyond these common targets, individual viruses showed distinct pathway specificity: DAV and DCV exhibited broad pathway targeting across multiple time points, Bloomfield virus affected broad pathways that decreased over time, while Nora virus only affected broad pathways at the mid–time point. DCV-infected animals at the mid-infection time point showed an aging acceleration that exceeded the effects attributable to any individual pathway or tissue studied (whole-genome aging ratio of 1.48 versus 0.86 to 1.14 in tissues and pathways respectively). This suggests that at this infection stage, DCV induces aging through different pathways and/or complex interactions among the aging hallmarks we examined.

To assess evolutionary conservation, we created aging references using only *Drosophila* genes with direct human orthologs. These human-ortholog-only references showed aging acceleration patterns largely concordant with the *Drosophila* whole-genome reference (last two rows Fig. 3), suggesting evolutionary conservation of virus-induced aging mechanisms and indicating the potential of these findings for understanding viral impacts on human aging and longevity.

To confirm that aging acceleration represents truly systemic effects rather than pathway-specific dysregulation, we performed exclusion analysis using aging references constructed from all genes except those associated with each pathway or tissue. When pathway-specific or tissue-specific genes were excluded from the aging references, we still detected comparable virus-induced aging acceleration across all time points and conditions. The exception was DCV at the mid-life time point, where epigenetic alterations and immune response genes were required to detect aging acceleration (fig. S5). This demonstrates that viral aging effects generally extend beyond the dysregulation of any single biological process or tissue, confirming that virus-induced aging represents a broad, systemic acceleration of biological age that cannot be attributed solely to targeted disruption of particular aging pathways.

### Viral load relationships suggest distinct aging mechanisms

**Fig. 4.**
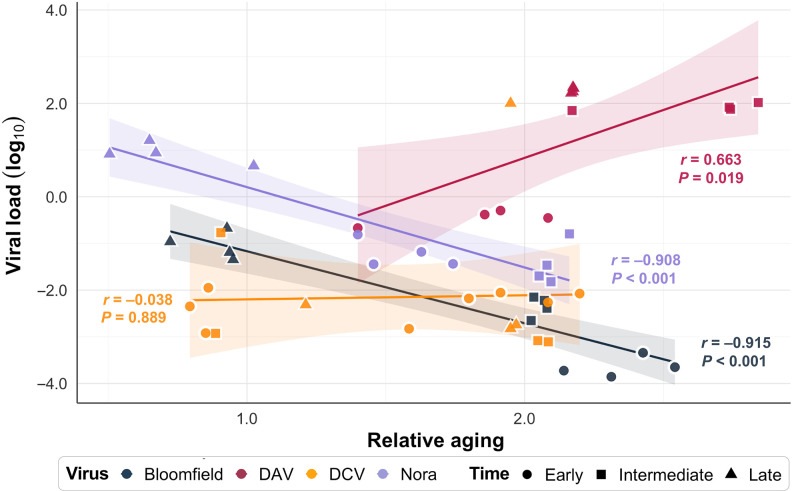
Correlation between relative aging (ratio between the biological and chronological age at a given time point) and viral load (viral RNA accumulation as determined by qRT-PCR) for four persistent viral infections in *Drosophila*. Early time points are 1 dpe (and 6 dpe for DCV), the intermediate time point is 12 dpe, and the late time point corresponds to 50% mortality of the host population. Solid lines represent linear regression fits for each virus. Shaded areas indicate 95% confidence intervals. Pearson correlation coefficients (*r*) and *P* values are displayed in color-matched text for each virus.

To explore if viral loads relate to aging acceleration, we evaluated correlations between virus RNA accumulation [measured by reverse transcriptase quantitative polymerase chain reaction (RT-qPCR) targeting the viral polymerase] and aging across the infection timeline (Fig. 4). The results showed a different relationship for each virus, suggesting distinct mechanisms of virus-induced aging.

DCV showed no relationship between virus levels and aging (Pearson’s *r* = −0.038, *P* = 0.889), indicating that aging acceleration occurs independently of how much virus accumulates in the host. DAV showed a strong positive correlation between virus levels and aging acceleration (*r* = 0.663, *P* = 0.019). This indicates that viral accumulation directly damages the host, with more virus accumulation causing proportionally more aging. Bloomfield and Nora viruses showed strong inverse relationships: Aging acceleration was greatest when virus levels were lowest (Bloomfield virus: *r* = −0.908, *P* < 0.001; Nora virus: *r* = −0.908, *P* < 0.001). This pattern suggests that the host’s efforts to fight these viruses drive aging. This counterintuitive pattern suggests that host defense responses may contribute to aging acceleration, although direct causality remains to be established. Overall, these findings combined with the virus-specific pathway targeting described above, demonstrate that virus-induced aging can occur through multiple mechanisms.

### Viral pathogenicity positively correlates with aging acceleration

Having established that less-lethal viruses produce smaller aging effects, we tested our hypothesis that viral pathogenicity (here defined as the reduction in host lifespan relative to uninfected controls) quantitatively predicts aging acceleration.

To establish the low end of the pathogenicity spectrum, we analyzed Orsay virus (OrV) infection in *Caenorhabditis elegans* larvae. OrV is an enteric RNA virus that infects nematodes with minimal pathogenicity (*45*, *46*). Using the transcriptional data of uninfected larvae (*47*), we established an aging reference using the same methodology described above. Using this reference, we observed that OrV infection had minimal impact on nematode aging. The biological age of infected nematodes tracks closely with chronological age except for a brief period of acceleration coinciding with peak viral replication [fig. S6, and Castiglioni *et al.* (*47*)]. This confirms that minimally pathogenic viruses cause overall negligible aging acceleration and provides a low-pathogenicity data point for correlation analysis.

**Fig. 5.**
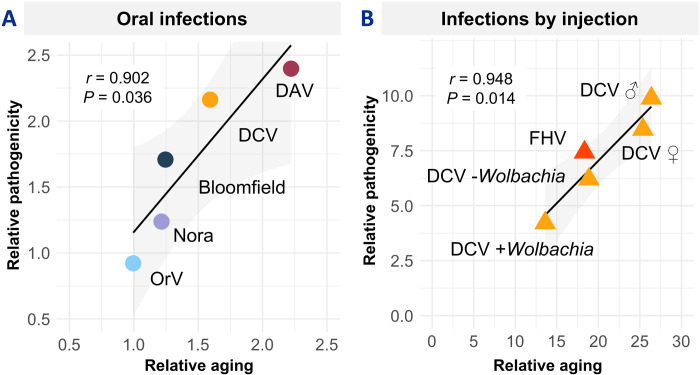
Viral pathogenicity correlates with aging acceleration across infection routes. Correlation between relative aging acceleration (ratio between the biological and chronological age) and relative pathogenicity (ratio between uninfected and infected host lifespan) for (**A**) Oral infections: Four *Drosophila* viruses spanning a wide pathogenicity range (DAV, DCV, Bloomfield virus, and Nora virus) plus nonpathogenic Orsay virus (OrV) in *C. elegans* larvae. Each point represents a different virus. (**B**) Injection infections: Correlation analysis extended to injection-delivered viruses, including DCV in male (♂) and female (♀) flies, flock house virus (FHV), and DCV in flies with (+*Wolbachia*) or without (−*Wolbachia*) symbiotic bacteria. Values >1.0 indicate increased mortality or aging acceleration relative to uninfected controls.

To quantify the relationship between pathogenicity and aging effects, we calculated relative pathogenicity (lifespan reduction) and relative aging acceleration using area under the curve calculations (see Materials and Methods). Across oral infections (four viruses in *Drosophila* and OrV in nematodes), we observed a significant positive correlation between infection pathogenicity and accelerated aging (Pearson’s *r* = 0.902, *P* = 0.036; Fig. 5A). This correlation suggests that aging acceleration is a key mechanism by which enteric viruses reduce host lifespan.

To test whether this relationship extends beyond natural oral infections, we examined viral infections initiated by injection. Here, injection bypasses gut barriers and typically results in increased viral pathogenicity. Using data from injected DCV (*48*, *49*) and flock house virus (FHV) (*50*), we found that aging was higher in injected animals and positively correlated with pathogenicity (Pearson’s *r* = 0.948, *P* = 0.014; Fig. 5B).

### Host factors modulate virus-induced aging

Having established virus-specific aging mechanisms, we next examined whether host characteristics could influence the magnitude of infection-induced aging. We tested various factors that could modulate virus-induced aging acceleration.

**Fig. 6.**
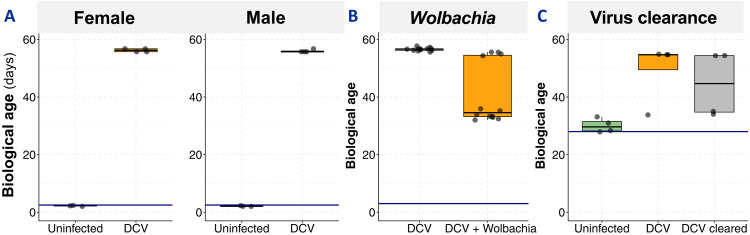
Host factors modulate virus-induced aging. Biological age estimates showing (**A**) sex-independent aging acceleration in DCV-infected flies, (**B**) *Wolbachia*-mediated protection against virus-induced aging, and (**C**) persistent aging effects in flies that cleared viral infection. Blue horizontal lines represent chronological age; boxplots show biological age distributions.

First, we examined host sex differences, as our initial analysis used female flies and *Drosophila* exhibits sex-specific aging pathology and transcriptional and immune responses (*35*, *51*–*53*). Using a transcriptional dataset that included DCV-infected males and females (*48*), we found comparable biological ages in uninfected animals of both sexes and similar significant acceleration of aging upon DCV infection (Fig. 6A).

Second, we studied the effect of *Wolbachia*, a common bacterial symbiont of insects known to reduce viral pathogenicity and transmission (*54*, *55*). Using a transcriptional dataset of flies injected with DCV and either harboring or not *Wolbachia* (*49*), we observed that harboring *Wolbachia* mitigated the aging induced by DCV. At 3 dpe, *Wolbachia*-free flies injected with DCV had a biological age of 56.7 ± 0.6 dpe (median ± SD). In contrast, flies harboring *Wolbachia* had a significant (Wilcoxon rank-sum test, *P* < 0.001) lower biological age of 34.6 ± 10.6 dpe (Fig. 6B).

Third, we examined viral clearance. Although viral infections are typically persistent in fly populations, some individuals can clear DCV infection (*41*, *56*). By examining data from flies persistently infected with DCV (*39*), we observed that both the DCV-infected flies and virus-cleared flies showed significantly higher biological age than uninfected flies; at 28 dpe the uninfected flies had a median biological age of 28.9 days, compared to 54.6 ± 10.4 dpe for the DCV-infected flies and 44.5 ± 11.4 dpe for flies that had cleared DCV infection (Fig. 6C). This suggests that once aging acceleration is triggered by viral infection, it persists even after viral clearance.

## DISCUSSION

Viruses have long been known to modulate their hosts in remarkable ways (*57*, *58*), but their role in modulating the aging process has only recently begun to be explored. Our work demonstrates that enteric viral infections modulate biological age in proportion to their pathogenicity, establishing viruses as quantifiable “age-distorters” that interfere with host aging trajectories (*59*).

Our findings reveal three distinct strategies by which viruses accelerate aging. DCV acts as an aging “switch” that triggers cellular aging programs at a magnitude that is independent of viral load, potentially explaining why mild chronic infections sometimes have disproportionate long-term consequences. The bimodal mortality distribution observed in DCV infection likely reflects this switch-like behavior. This interpretation is supported by pathway-specific aging analysis, where DCV at mid-infection shows whole-genome aging acceleration exceeding that of any single pathway examined, suggesting activation of complex or threshold-dependent aging programs that vary among individuals. DAV causes dose-dependent aging where higher viral loads produce proportionally greater aging acceleration, indicating direct pathogen-mediated damage. Nora and Bloomfield viruses show inverse correlations where aging peaks during viral suppression by the immune system, suggesting that host defense responses can drive aging more than the pathogens themselves.

Evolutionary conservation strongly suggests translational relevance. Most aging-associated gene families originated hundreds of millions of years ago (*60*). These ancient pathways represent universal targets, as viral proteins have convergently evolved to directly interact with host aging networks across phylogenetically distant species (*61*). Consistent with this deep conservation, both universal and species-specific aging mechanisms operate across diverse organisms from flies to mammals (*62*), indicating that viral modulation of aging likely targets both conserved pathways and species-specific mechanisms. We observe consistent acceleration of aging in gut and fat body tissues, which parallels intestinal and metabolic dysfunction in mammalian aging (*63*). In addition, host-associated microorganisms have been shown to modulate viral infections and transmission in diverse hosts (*64*–*66*). We demonstrate that *Wolbachia*’s protective effects extend to mitigation of virus-induced aging, showing that beneficial microorganisms can modulate infection-induced aging effects.

Methodological robustness enables broad adoption. Our aging clock framework demonstrates consistent performance across different datasets, fly strains, and experimental conditions, aligning with RAPToR’s demonstrated compatibility between datasets even though optimal results are achieved within datasets (*19*). Pathway-specific analyses confirm that viruses induce genuine aging perturbation. Hence, our framework and dataset can be widely adopted for aging research in *Drosophila* and potentially other organisms.

Persistent aging effects require further investigation. Flies that successfully cleared infections retained elevated biological age to varying degrees, indicating that aging acceleration involves fundamental cellular changes beyond transient physiological disruption. Our observations align with findings in humans, where irreversible changes in the epigenome and immune system have been observed following clearance of viral infections (*67*, *68*). Similarly, clearance of chronic hepatitis C virus only partially reversed aging in some individuals while having no effect in others (*69*). This persistent effect suggests that targeted interventions may be needed beyond simply supporting viral clearance.

Future directions include identifying molecular mechanisms by which different viruses target aging pathways and testing whether established anti-aging interventions can prevent or reverse virus-induced aging acceleration. The framework should be extended to DNA viruses, bacteria, and fungi to reveal general principles of microbe-host aging interactions. Clinically, transcriptional aging clocks could complement traditional markers by revealing long-term health impacts not apparent through conventional assessments, particularly for infections causing mild, short-term symptoms but substantial aging acceleration. The discovery that some microorganisms can slow aging opens therapeutic possibilities for developing beneficial microorganisms as aging interventions, although such approaches require rigorous safety evaluation. Our findings on virus-induced aging provide a foundation for investigating aging mechanisms, identifying anti-aging targets, reducing infection pathogenicity, and leveraging viruses as tools to modulate aging.

### Limitations and alternative interpretations of the present study

While our framework provides a robust quantitative measure of virus-induced aging, the findings should be considered in the context of ongoing uncertainty surrounding the biological interpretation of transcriptional aging clocks. These models may either register downstream manifestations of aging or, alternatively, capture features more closely linked to its underlying causal architecture. We anticipate that the present results will contribute meaningful evidence toward resolving this debate and offer a more grounded basis for interpreting transcriptional age in a biological context.

First, in analyses designed to estimate the overall impact of infection on biological age, it is necessary to use summarizing metrics such as the area under the disease progress stairs (*70*). While valuable for capturing this cumulative effect, these metrics intrinsically condense complex temporal fluctuations and may oversimplify the distinct modes of acceleration and deceleration occurring between sampling time points.

Second, population heterogeneity presents methodological challenges, particularly in infections exhibiting bimodal distributions, such as DCV. Although investigating the early- versus late-succumbing subpopulations independently would provide valuable insights, we currently lack reliable nonlethal phenotypic markers of imminent death for this viral infection. For instance, we unsuccessfully attempted the Smurf assay in DCV-infected populations: The Smurf phenotype does not manifest in early-succumbing individuals, likely because DCV-induced mortality primarily stems from intestinal obstruction (*71*) rather than increased barrier leakage. This rendered us unable to sort near-death populations before transcriptomic analysis. We observed similar intrinsic phenotypic variability in the biological age of the *Wolbachia* cohorts. According to the authors (*49*) these populations were established under identical conditions and shared the same host genetics (*w^1118^*), *Wolbachia* strain (*wMelCS_b*), viral exposure, and husbandry. Therefore we performed whole-population analyses, which demonstrated a significant overall mitigation of virus-induced aging. However, we observed a subset of individuals where this protective effect was markedly stronger. This heterogeneity could arise from stochastic biological variation, divergence within-host infection dynamics despite synchronized virus inoculation, or complex host-symbiont-virus interactions that warrant future investigation.

Third, the apparent transcriptomic “rejuvenation” (where biological age falls below chronological age) observed in late-stage survivors of milder infections, such as Bloomfield and Nora viruses, presents a fascinating paradox. Because approximately half of the infected population has already died by the late time points (36 and 52 dpe from Bloomfield and Nora virus, respectively), the reduced biological age observed in the remaining flies could represent a survival selection bias, wherein only the most resilient individuals that have successfully deployed tolerance mechanisms are being quantified. We hypothesize that in these more tolerant individuals, mild viral damage actively mitigates the aging process through early epithelial remodeling. Bloomfield and Nora induce a modest, yet significant, early activation of ISC proliferation compared to uninfected controls (*37*). Qin *et al.* (*72*) demonstrated that inducing low-level early midgut injury (via transient chemical stress or targeted genetic ablation) triggers controlled enterocyte turnover. In a standard midgut, enterocytes are generated synchronously during development and undergo a catastrophic, collective die-off during aging. However, early injury forces asynchronous cell replacement, embedding newly differentiated cells among older ones to create a protective “epithelial age mosaic.” This asynchronous cellular mosaic prevents the abrupt, synchronized collapse of the intestinal barrier later in life. We hypothesize that in flies capable of tolerating a mild persistent infection, the virus serves as the initial stressor driving this mosaicism. Consequently, once the host passes a critical survival threshold, the remodeled gut epithelium effectively decelerates the host’s long-term biological aging trajectory.

Last, while the field continues to pursue increasingly refined aging markers, transcriptomic clocks remain robust tools to advance our understanding of biological aging. In the context of viral infections, distinguishing genuine aging responses from immune ones is critical. Our exclusion analyses and the detection of validated physiological markers, such as increased ISC proliferation, confirm that the estimated age acceleration reflects a true aging process rather than an immune artifact, supporting the notion that virus-induced aging signatures capture genuine physiological frailty. Future work integrating emerging phenotypic markers with higher-resolution temporal sampling will be essential to fully resolve the intersection of viral pathogenesis, immune dynamics, and biological aging.

## MATERIALS AND METHODS

### Dataset biological information

All RNA samples come from whole flies or nematodes. Unless otherwise indicated, all flies are *Wolbachia*-free and mated *D. melanogaster*. Persistent infected flies data belong to four pools of five *w^1118^* flies per virus and time point (*41*). DCV male and female dataset consists of four pools of 4 to 7 dpe 10 Oregon-RT virgin flies per condition (*48*). DCV and *Wolbachia* dataset consists of 12 pools of 6 to 9 dpe 10 *w^1118^* flies per condition (*49*). FHV dataset has four measures per condition of an undetermined number of 6 to 9 dpe Oregon-R male flies (*50*). OrV dataset has three pools of 1200 *C. elegans* nematodes per condition (*47*).

### Transcriptomic data

Persistent infected flies’ raw sequencing reads can be found in the Sequence Read Archive under the BioProject PRJNA1235228. For injected flies, raw sequencing reads were retrieved from the same archive: FHV from BioProject PRJNA644593, DCV and *Wolbachia* from BioProject PRJEB21984, DCV in male and female from BioProject PRJNA1084779, and Smurf flies from BioProject PRJNA906973. The *C. elegans* mapped reads were provided by the laboratory of S. F. Elena. Raw sequencing reads were mapped to the *D. melanogaster* genome release dmel_r6.56 with STAR (version 2.7.11b) (*73*). Feature counting was done with HTSeq (version 0.11.2) (*74*) using the default settings.

### Biological categories

To categorize the biological processes affected during aging and viral infections, we established categories to capture major cellular and physiological processes. On the basis of gene ontology (GO) terms, these categories encompass: Immune defense (GO terms containing keywords such as immune, antimicrobial, antiviral, defense, inflammatory, pathogen, bacteria, and virus), transport and trafficking (transport, localization, secretion, vesicle, trafficking, endocytosis, and exocytosis), cell cycle and division (cell cycle, mitosis, division, proliferation, DNA replication, and chromosome), gene expression (transcription, translation, expression, RNA, protein, gene regulation, and chromatin), development and differentiation (development, morphogenesis, differentiation, organogenesis, embryonic, and larval, growth), cell signaling (signal, signaling, response, communication, receptor, kinase, and phosphorylation), cell death and apoptosis (death, apoptosis, necrosis, autophagy, and programmed cell death), metabolic processes (metabolic, metabolism, catabolism, anabolism, biosynthetic, glycol, lipid, carbohydrate, amino acid, and energy), and reproduction (reproduction, sex, mating, gamete, oogenesis, and spermatogenesis).

### Age estimation

We estimated biological age from transcriptomic data using the RAPToR package (version 1.2.0) (*19*) in R (version 4.3.2) in the RStudio development environment (version 2024.04.2+764). First, a reference was created from uninfected flies collected on days 1, 6, 12, 25, 28, 36, 52, and 58 posteclosion [Castelló-Sanjuán *et al.* (*41*)]. Gene expression counts were fitted to a generalized additive model via RAPToR’s (version 1.2.0) ge_im function using a cubic regression spline with eight knots. We then performed a PCA with the prcomp function in the “stats” package, determining that 29 principal components should be retained (fig. S7) by summing those with a cumulative variance below 0.99 and then adding one. Following RAPToR methodology, we included components with intelligible dynamics. As a control for overfitting, using only the first 10 components provided similar results (fig. S8). This approach leverages RAPToR’s reference interpolation capability, which decomposes the expression matrix into components that summarize gene expression dynamics and interpolates these components with respect to time to reconstruct full interpolated gene expression profiles. The final reference was constructed using RAPToR’s make_ref function with 1000 interpolation points. Subsequently, we estimated the biological ages of additional transcriptomic datasets by applying RAPToR’s ae function to this reference. In specific cases described in the main text, new references were generated using the same approach but were restricted to subsets of genes of interest (file S1) derived from the original uninfected dataset. For analyses involving virgin fly datasets (*48*), genes that are differentially expressed after mating (*75*) were excluded from reference construction to avoid mating status confounding effects (file S3).

### Pathway- and tissue-specific gene sets curation

Aging hallmark genes were curated from established frameworks (*15*) and cross-referenced with FlyBase Gene Ontology annotations (release FB2024_02). Pathways were defined using the following GO terms: genomic instability: DNA damage response (GO:0006974); telomere attrition: telomere maintenance (GO:0000723); epigenetic alterations: chromatin organization (GO:0006325); loss of proteostasis: response to topologically incorrect protein (GO:0035966) and protein ubiquitination (GO:0016567); disabled macroautophagy, autophagy (GO:0006914) and protein targeting to vacuole (GO:0006623); deregulated nutrient sensing: response to insulin (GO:0032868), TOR signaling (GO:0031929), response to starvation (GO:0042594), and lipid metabolic process (GO:0006629); mitochondrial dysfunction: mitochondrion organization (GO:0007005) and electron transport chain (GO:0022900); cellular senescence: cellular senescence (GO:0090398); stem cell exhaustion: stem cell population maintenance (GO:0019827); altered intercellular communication: signaling (GO:0023052); and immune response: immune response (GO:0006955), defense response (GO:0006952), defense response to other organism (GO:0098542), and response to bacterium (GO:0009617).

Tissue-enriched genes were obtained from FlyBase tissue ontology annotations using the following anatomical terms: ovary (FBbt:00004865), adult somatic muscle (FBbt:00058784), fat body (FBbt:00005066), adult gut (FBbt:00007513), and brain (FBbt:00005095). The number of genes per pathway and tissue are indicated in Fig. 3, with complete gene lists provided in file S2.

### Viral load quantification

Our experimental design uses persistent viral infections established through natural oral acquisition during larval development. Viral loads in these infections are not experimentally imposed but reflect endogenous steady-state levels maintained during persistent infection. Our previous work [Castelló-Sanjuán *et al.* (*41*)] demonstrated relatively homogeneous viral loads within each virus type across individuals, with the exception of DCV, which shows greater interindividual variation. All samples analyzed in this study were confirmed to carry comparable viral loads within each virus type (file S1). These steady-state loads represent biologically relevant infection intensities that reflect equilibrium between viral replication and host antiviral responses.

Viral loads were measured by RT-qPCR in our previous work [Castelló-Sanjuán *et al.* (*41*)]. Individual flies were homogenized in TRIzol for RNA extraction, followed by cDNA synthesis using random primers and qPCR using virus-specific primers. Viral RNA levels were normalized to the housekeeping gene *Rp49* and calculated as 2^−ΔCt^ values, with a detection threshold set at 35 Ct cycles (samples with Ct > 35 were considered uninfected). For each sample, since each sample was prepared by pooling equal RNA amounts from five individuals, we calculated the mean viral load from the five corresponding individual flies measured separately by RT-qPCR (file S1).

### Calculating relative aging and pathogenicity

Ratios were calculated by first summarizing the time series of estimated biological age and survival curves (file S1) into a single value using the area under the disease progress stair (AUDPS) calculation (*70*). These values were then used to calculate the relative aging or relative pathogenicity by dividing the AUDPS of the infected samples (either for aging or survival) by the AUDPS of their uninfected controls. The AUDPS calculation was performed using the “agricolae” package (version 1.3-7) in R (version 4.3.2). Survival data were extracted using computer vision assist (automeris.io) from survival plots already published: fly persistent infections come from Castelló-Sanjuán *et al.* (*41*), FHV from Sheffield *et al.* (*50*), DCV-injected male/female from Salminen *et al.* (*48*), DCV-injected with or without *Wolbachia* from Martinez *et al.* (*49*), and OrV from Ashe *et al.* (*46*).

### Statistical analysis

Pearson correlations were calculated in R (version 4.3.2) using the cor.test function from the “stats” package.
