# Supplementary material for: Enteric viral infections promote systemic accelerated aging in Drosophila
Source: Sci Adv. 2026 Jun 5;12(23):eaec1735. doi: 10.1126/sciadv.aec1735 (PMC13240232; doi:10.1126/sciadv.aec1735)
Supplement: Supplementary file 1 — Figs. S1 to S8 Legends for files S1 to S5 [file sciadv.aec1735_sm.pdf]

Science Advances
AAAS

Supplementary Materials for

Enteric viral infections promote systemic accelerated aging in *Drosophila*

Rubén González, *et al.*

Corresponding author: Rubén González, ruben.gonzalez-miguelez@pasteur.fr;
Maria-Carla Saleh, carla.saleh@pasteur.fr

Sci. Adv. **12**, eaec1735 (2026)
DOI: 10.1126/sciadv.aec1735

The PDF file includes:

Figs. S1 to S8
Legends for files S1 to S5

Other Supplementary Material for this manuscript includes the following:

Files S1 to S5

SUPPLEMENTARY FIGURES

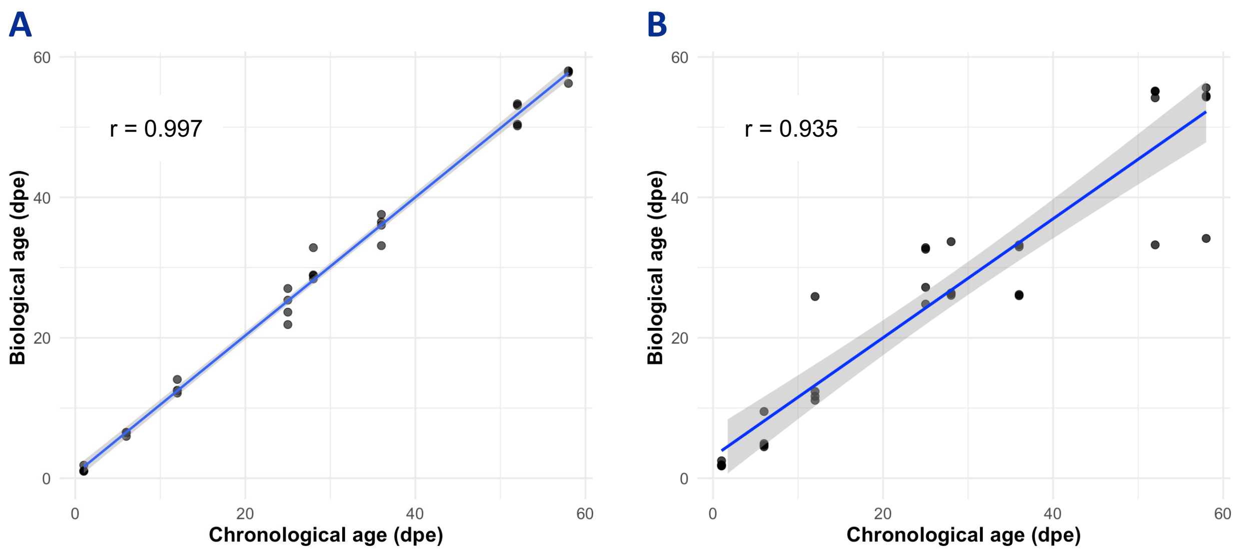

Fig. S1. Validation of RAPToR-based biological age estimation in *Drosophila* reference dataset. (A) Full reference performance showing the correlation between chronological age and RAPToR-estimated biological age when all 32 samples (four biological replicates at each one of the eight timepoints: (1, 6, 12, 25, 28, 36, 52, and 58 days post-eclosion, dpe) are included in both reference construction and age estimation (r = 0.997). **(B)** Leave-one-out cross-validation results showing the correlation between chronological age and RAPToR-estimated biological age when each sample is individually excluded from reference construction and then predicted using the remaining 31 samples (r = 0.935). Each point represents one of the 32 individual predictions, with colors indicating different biological replicates. The blue line represents the linear regression fit with 95% confidence interval (gray shading).

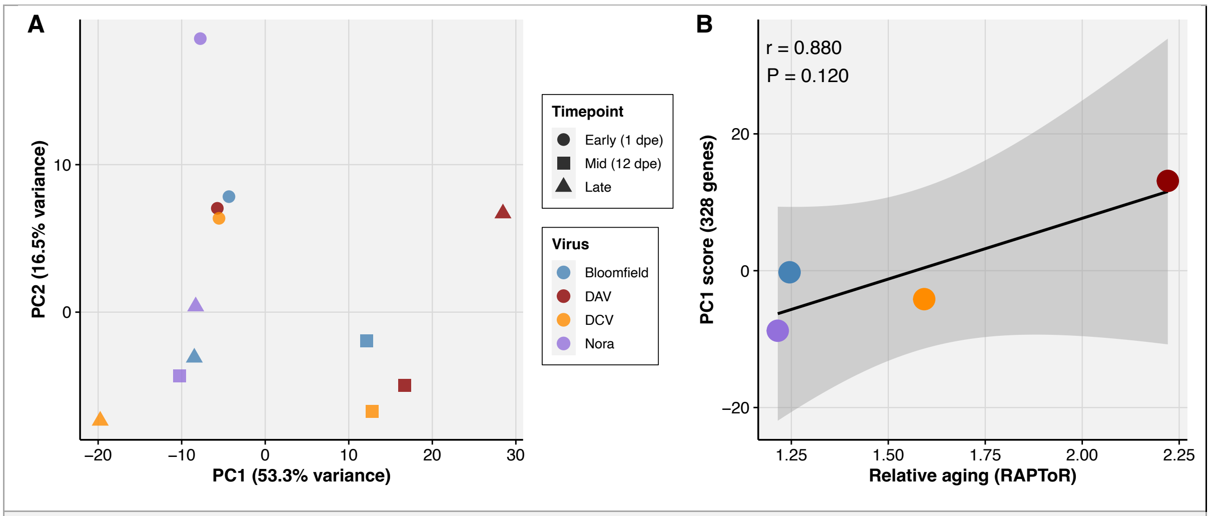

Fig. S2. Chronological age-correlated genes from dying flies show positive correlation with aging. (A) PCA visualization of virus-infected samples using the 328 chronological age-correlated genes identified by Scott and colleagues (2025). Points represent individual samples colored by virus (DAV, DCV, Bloomfield, Nora) and shaped by life timepoint (Early: 1 dpe, Mid: 12 dpe, Late: 50% virus mortality). PC1 and PC2 account for 53.3% and 16.5% of variance, respectively. **(B)** Correlation between PC1 scores (averaged per virus across timepoints) and RAPToR-based aging acceleration. Black line shows linear regression with 95% CI. The positive correlation (Pearson's r = 0.880, *P* = 0.120) validates that dying-phase chronological markers correlate with cumulative aging, though these metrics capture distinct temporal aspects of the aging process.

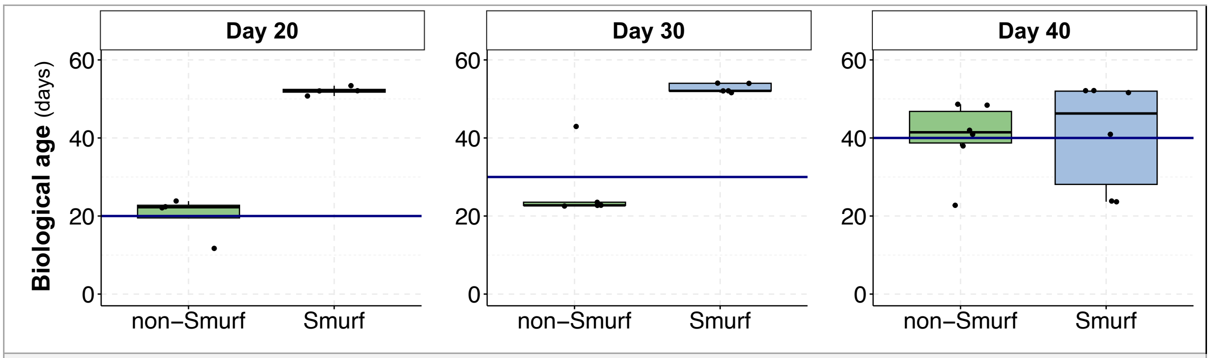

Fig. S3. Transcriptional aging clock captures physiological frailty in the terminal pre-death phase. Biological age estimates for uninfected flies stratified by Smurf status at three chronological ages (20, 30, and 40 dpe). Non-Smurf flies (green) maintain biological ages close to chronological age, while age-matched Smurf flies (blue) show markedly accelerated aging at all timepoints, validating that the aging clock captures physiological frailty states. Blue horizontal lines represent chronological age; boxplots show biological age distributions. Data from Zane et al. (44).

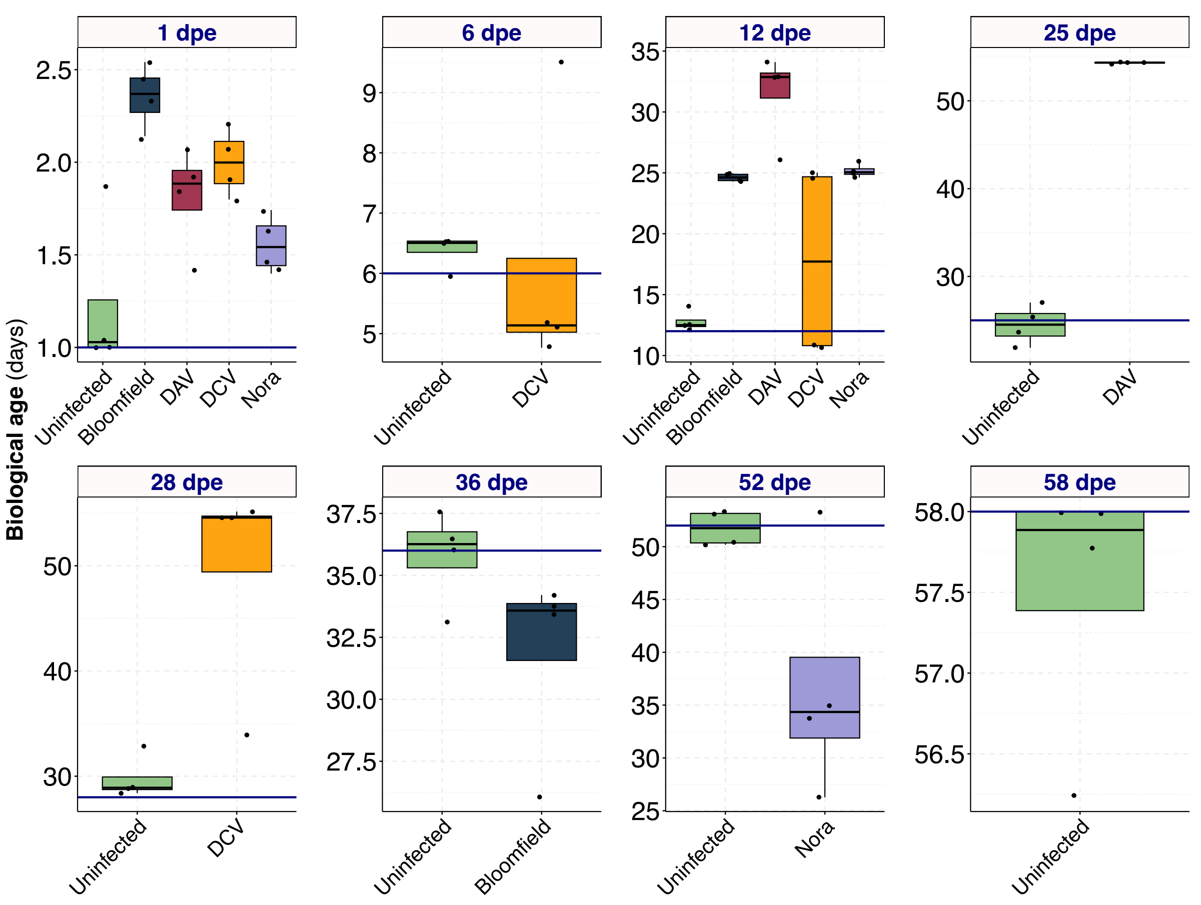

Fig. S4. Box plot representation of the biological age of persistently infected and uninfected flies from Figure 2. Each panel represents a specific chronological timepoint, with the horizontal line indicating the chronological age of the samples.

	Reference	Early (1 dpe)					Mid (12 dpe)					Late (50% mortality)				
		Uninfected	DAV	DCV	Bloomfield	Nora	Uninfected	DAV	DCV	Bloomfield	Nora	Uninfected	DAV	DCV	Bloomfield	Nora
Pathway	Altered signaling (excluded) (n=15808)	1.03	2.17	1.94	2.23	1.51	1.03	2.45	1.61	2.22	2.43	0.99	2.17	1.94	0.86	0.63
Pathway	Nutritional status (excluded) (n=16824)	1	2	2.08	2.4	1.57	1.02	2.51	1.56	2.24	2.39	1	2.17	1.95	0.86	0.63
Pathway	Immune response (excluded) (n=16963)	1	1.74	1.97	2.26	1.48	1.03	2.58	0.97	2.35	2.41	1	2.15	1.94	0.86	0.6
Pathway	Epigenetic alterations (excluded) (n=17146)	1.03	1.88	1.97	2.23	1.37	1.02	2.49	0.98	2.21	2.29	1	2.16	1.94	0.87	0.66
Pathway	Mitochondrial dysfunction (excluded) (n=17300)	1.03	1.86	2.11	2.31	1.57	1.02	2.47	1.54	2.21	2.19	1	2.17	1.94	0.86	0.66
Pathway	Genomic instability (excluded) (n=17345)	1.03	1.91	1.94	2.31	1.34	1.02	2.49	1.55	2.21	2.26	1	2.17	1.94	0.88	0.66
Pathway	Loss of proteostasis (excluded) (n=17364)	1.03	1.86	2.08	2.34	1.43	1.02	2.53	1.54	2.21	2.18	1	2.17	1.94	0.87	0.66
Pathway	Disabled macroautophagy (excluded) (n=17379)	1.03	1.88	2.08	2.37	1.48	1.02	2.53	1.54	2.21	2.23	1	2.17	1.95	0.86	0.66
Pathway	Stem Cell Exhaustion (excluded) (n=17504)	1.03	1.88	2.06	2.4	1.46	1.02	2.49	1.55	2.21	2.26	1	2.16	1.95	0.87	0.66
Pathway	Telomere attrition (excluded) (n=17578)	1.03	1.83	2.11	2.31	1.48	1.02	2.53	1.54	2.21	2.23	1	2.17	1.95	0.86	0.66
Tissue	Brain (excluded) (n=15754)	1.03	2.11	1.83	2.2	1.6	1.03	2.38	1.6	2.24	2.38	0.99	2.16	1.94	0.87	0.65
Tissue	Ovary (excluded) (n=16639)	1.09	2.08	2.14	2.51	1.91	1.02	2.36	2.16	2.23	2.17	1	2.16	1.94	0.83	0.61
Tissue	Fat body (excluded) (n=17011)	1	1.88	2	2.14	1.4	1.03	2.62	1.55	2.18	2.14	1	2.18	1.94	0.87	0.63
Tissue	Adult gut (excluded) (n=17457)	1.03	1.91	2.08	2.23	1.54	1.02	2.5	1.56	2.22	2.27	1	2.17	1.94	0.86	0.66
Tissue	Adult somatic muscle (excluded) (n=17509)	1.03	2.08	2.17	2.4	1.63	1.02	2.47	1.56	2.21	2.16	0.99	2.17	1.95	0.86	0.65
	Human homologs (excluded) (n=8515)	1.03	2.45	2.4	2.71	2.2	1.02	2.52	2.47	2.44	2.47	1	1.2	1.49	0.85	0.59
	**Drosophila whole genome reference** (n=17610)	1.03	1.88	2	2.37	1.54	1.04	2.74	1.48	2.05	2.09	1.01	2.17	1.95	0.93	0.66

Relative aging (biological / chronological age): 1 2 3 4 5

Fig. S5. Exclusion analysis. Heatmap shows median relative aging acceleration (biological age/chronological age) using aging references constructed by excluding specific pathway or tissue genes. Early life (1 dpe), mid-life (12 dpe), and late life timepoints (DAV: 25 dpe, DCV: 28 dpe, Bloomfield virus: 36 dpe, Nora virus: 52 dpe, representing ~50% population mortality). Values >1.0 (red) indicate accelerated aging, values <1.0 (green) indicate slowed aging, and values 1.0 (white) indicate normal aging progression. Numbers in parentheses indicate the number of genes in each reference set. Median absolute deviation values provided in Supplementary File 1.

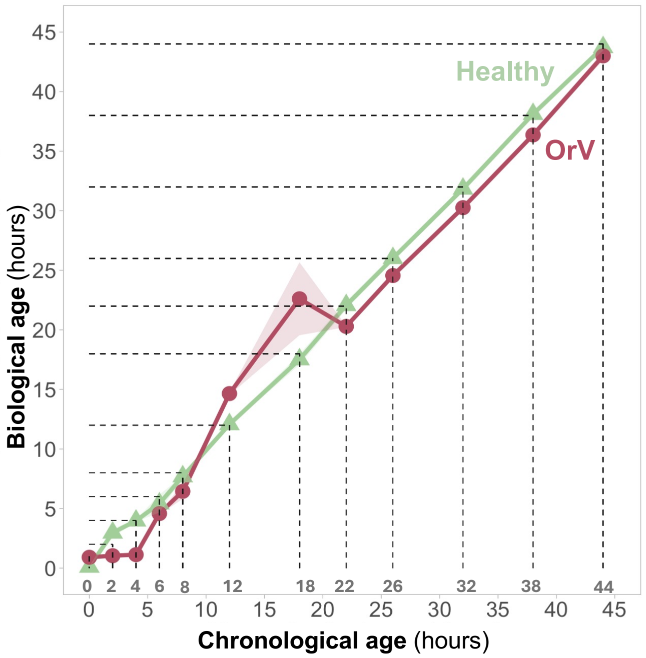

Fig. S6. *C. elegans* biological age (Y-axis) at different chronological timepoints (X-axis) for uninfected animals (green triangles) and those infected with Orsay virus (red circles). Points represent the mean biological age, and the shaded area represents the standard error.

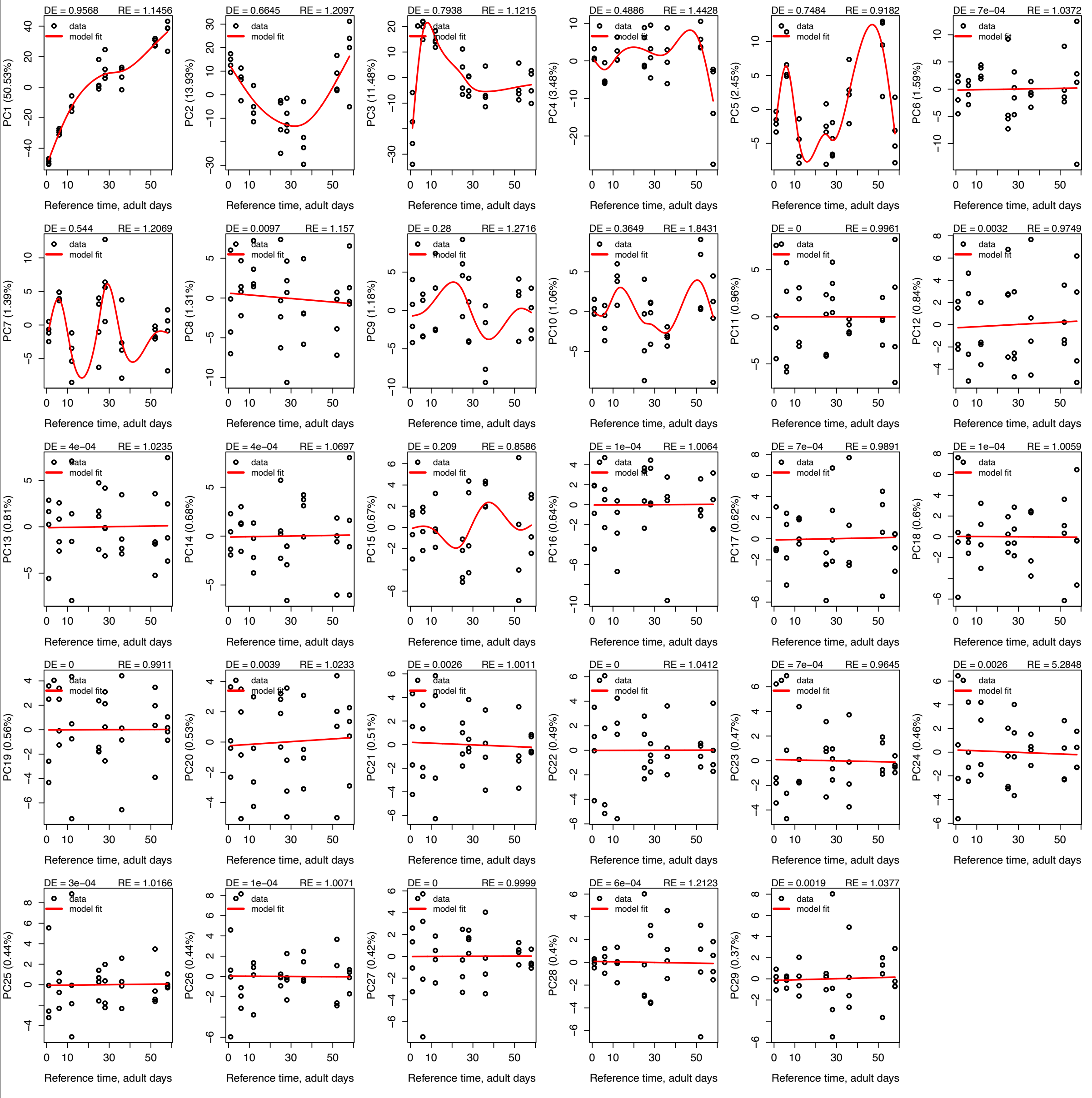

Fig. S7. Graphs with the 29 Principal Component Analysis (PCA), which decompose the expression matrix into components, used to create the adult fly reference.

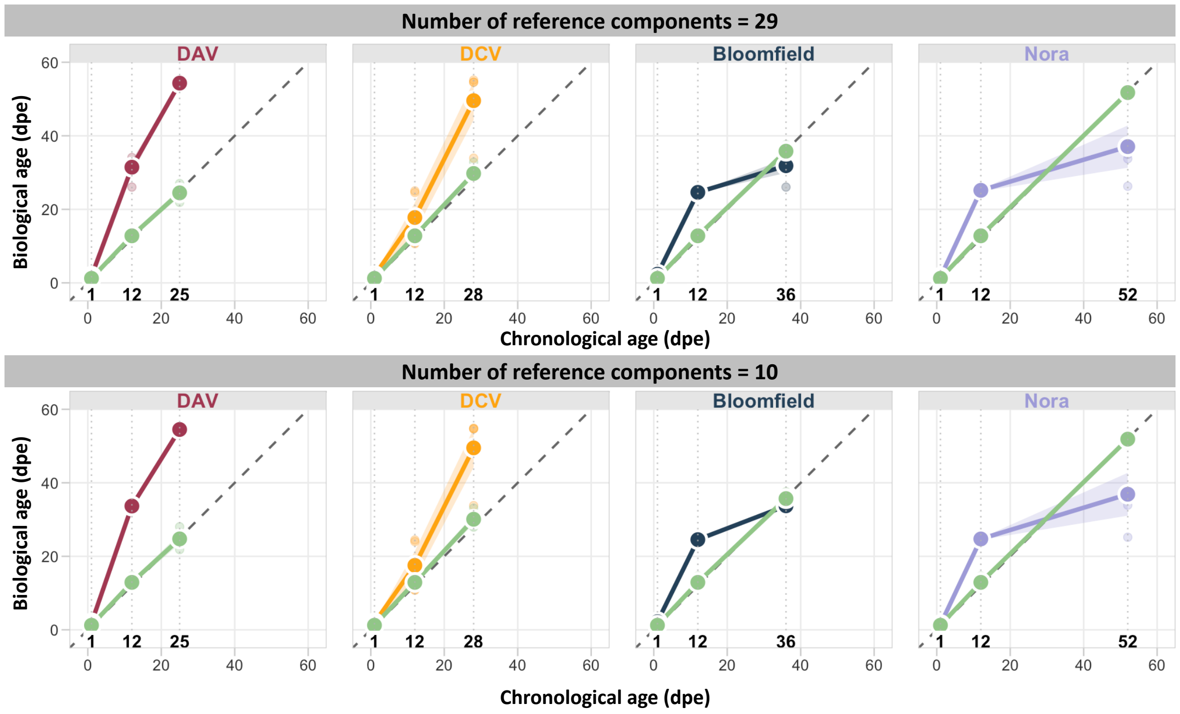

Fig. S8. RAPToR biological age estimation is robust to the number of principal components used in reference construction. Comparison of biological age estimates using RAPToR references constructed with either 29 principal components (top panel) or 10 principal components (bottom panel) as a control for overfitting. Each subplot shows the correlation between chronological age and estimated biological age for different viral infections: DAV (red), DCV (orange), Bloomfield virus (blue), and Nora virus (purple), compared to uninfected controls (green). The dashed diagonal line represents perfect prediction (y = x). Both approaches yield highly similar results across all conditions, demonstrating that the aging reference is robust and not subject to overfitting when using the full 29-component model.

SUPPLEMENTARY FILES

Supplementary File 1. Data generated per figure.

Supplementary File 2. List of pathway and tissue-specific aging genes.

Supplementary File 3. List of genes differentially expressed after mating (Newell et al. (75)) excluded from the reference to create a reference for virgin flies.

Supplementary File 4. R script detailing the generation of the *Drosophila* aging reference and subsequent calculation of transcriptomic biological age.

Supplementary File 5. Mapped RNA-seq counts from Castelló-Sanjuán et al. (41) utilized for *Drosophila* reference construction and biological age estimation.
